# Editorial: Non-human Primate Models of Psychiatric Disorders

**DOI:** 10.3389/fnbeh.2021.774064

**Published:** 2021-11-12

**Authors:** Rafael S. Maior, Hisao Nishijo, Fabio V. Caixeta

**Affiliations:** ^1^Department of Physiological Sciences, Institute of Biological Sciences, University of Brasilia, Brasilia, Brazil; ^2^System Emotional Science, University of Toyama, Toyama, Japan

**Keywords:** primate, psychiatric disorder, animal models, behavior, translational studies

As our civilization faces the complex social and health challenges of the twenty-first century, the quest for scientific progress seems more critical than ever before. The field of Biomedical research, in particular, is required to balance this pressing demand for knowledge with ethical constraints on both human and animal research. This issue raises fierce debates on the relevance of animal models for the advancement of science and society well-being, going as far as questioning if current animal research is still ethical, or even necessary. Although the reasoning for pursuing ethical experimentation on animal subjects is obvious for researchers in the field, the public discourse is often obscured by science illiteracy and misinformation. Ethical debate over the justification of animal research is particularly controversial in the case of non-human primates (NHP).

Despite steady progress in computational techniques, *in-silico* models, and transgenic rodent models, all of these approaches are still far from replacing NHPs. Translational methods for Psychiatric research should produce results which are, at the very least, predictive of one or more clinical outcomes. In this regard, NHPs are by far best suited to model psychiatric symptoms. The phylogenetic proximity to humans translates into more similar physiological and behavioral profiles, social structures, and developmental progress. For instance, NHPs are reliably used in addiction studies [see Maior (2011)] as they show reactions to those observed in humans during abstinence (Weerts et al., 2007). Also, very specific and complex responses, such as hallucinatory behavior, can be used as behavioral parameters in NHP (e.g., Ellison et al., 1981; Castner and Goldman-Rakic, 2003).

In this sense, the present topic gathered a small but very representative sample of studies which underscores the importance of NHP research for Psychiatry and Biomedicine. It includes diverse techniques and strategies to explore neurobiological disorders, ranging from stress and anxiety to schizophrenia and addiction. Natural drives and experimental evidence reveals marmosets as hitherto unappreciated models for research on psychiatric disorders related to stress de Sousa et al., while Novak and Meyer evaluate the existing evidence and future perspectives on marmoset models of depression, presenting a detailed review of self-injury behavior and discussing the prospects of this model for non-suicidal self-injury in human patients. Feng et al. reviewed the state-of-the-art regarding models of autism and their relationship with sleep disorders. In order to elucidate physiological aspects of addiction-related behaviors, Daddaoua et al. performed a detailed behavioral testing of decision-making strategies that underlie contingency management in macaques, bearing important perspectives for future addiction treatment in humans. Rowland et al. used magnetoencephalography in macaques to detail resting-state brain networks prior and post exposure to alcohol. Based on these data, they propose specific network characteristics which could predict high-risk of drinking phenotype. Costa et al. analyzed the feasibility of a translational model of schizophrenia employing visual illusions to probe the effects of glutamatergic antagonism. By means of single unit electrophysiological recordings, Dinh et al. explored the role of the prefrontal cortex in the processing of threatening stimuli and put forward a working hypothesis of how this structure may be involved in the origin of ophidiophobia. Waguespack et al. used intracerebral infusions in awake macaques to delineate the differences in brain circuitry related to sensory-gated responses between rodents and primates. Finally, Labuguen et al. present an important refinement to behavioral analyses of primates by providing open dataset of macaques which can be used for improving neural networks in motion capture tasks.

These studies outline the breadth of NHP research, and offer a valuable snapshot of how current primate research, using both classical and state-of-the-art techniques, continue to yield valuable insights into pathophysiological aspects of psychiatric conditions in the 2020's.

## Author Contributions

RM wrote initial draft. HN and FC edited and presented the final form. All authors contributed to the article and approved the submitted version.

## Conflict of Interest

The authors declare that the research was conducted in the absence of any commercial or financial relationships that could be construed as a potential conflict of interest.

## Publisher's Note

All claims expressed in this article are solely those of the authors and do not necessarily represent those of their affiliated organizations, or those of the publisher, the editors and the reviewers. Any product that may be evaluated in this article, or claim that may be made by its manufacturer, is not guaranteed or endorsed by the publisher.
